# Occupational anaphylaxis: A Position Paper of the German Society of Allergology and Clinical Immunology (DGAKI) 

**DOI:** 10.5414/ALX02543E

**Published:** 2024-11-28

**Authors:** Regina Treudler, Margitta Worm, Andrea Bauer, Heinrich Dickel, Guido Heine, Uta Jappe, Ludger Klimek, Monika Raulf, Bettina Wedi, Dorothea Wieczorek, Wojciech Francuzik, Thilo Jakob, Oliver Pfaar, Johannes Ring, Franziska Rueff, Sabine Schnadt, Thomas Werfel, Gerda Wurpts, Julia Zarnowski, Torsten Zuberbier, Knut Brockow

**Affiliations:** 1 Institute of Allergology, Charité – Universitätsmedizin Berlin, Corporate Member of Freie Universität Berlin and Humboldt-Universität zu Berlin, Berlin,; 2 Department of Dermatology, Venereology and Allergology, Charité – Universitätsmedizin Berlin, Corporate member of Freie Universität Berlin and Humboldt-Universität zu Berlin, Berlin,; 3 Department of Dermatology, University AllergyCenter, University Hospital Carl Gustav Carus, Technical University Dresden, Dresden,; 4 Department of Dermatology, Venereology and Allergology, St. Josef Hospital, University Medical Center, Ruhr University Bochum, Bochum,; 5 Department of Dermatology, Venereology and Allergy, University Hospital Schleswig-Holstein, Campus Kiel, Kiel,; 6 Division of Clinical and Molecular Allergology, Research Center Borstel, Airway Research, Center North (ARCN), Member of the German Center for Lung Research, Borstel, Interdisciplinary Allergy Outpatient Clinic, Department of Pneumology, University of Luebeck,; 7 Department of Otolaryngology, Head and Neck Surgery, Universitätsmedizin Mainz, Mainz and Center for Rhinology and Allergology, Wiesbaden,; 8 Department of Allergology/Immunology; Institute for Prevention and Occupational Medicine of the German Social Accident Insurance, Institute of the Ruhr-University Bochum (IPA), Bochum, Germany,; 9 Department of Dermatology and Allergy, Comprehensive Allergy Center, Hannover Medical School, Hannover,; 10 Department of Dermatology and Allergology, University Medical Center Giessen (UKGM), Justus-Liebig-University Giessen, Giessen,; 11 Department of Otorhinolaryngology, Head and Neck Surgery, Section of Rhinology and Allergy, University Hospital Marburg, Philipps-Universität Marburg, Marburg,; 12 Department of Dermatology and Allergy Biederstein, School of Medicine and Health, Technical University of Munich TUM),; 13 Department of Dermatology and Allergy, University Hospital, LMU Munich, Munich,; 14 German Allergy and Asthma Association (DAAB), Mönchengladbach,; 15 Clinic for Dermatology and Allergology, Aachen Comprehensive Allergy Center (ACAC), University Hospital of RWTH Aachen University, Aachen,; 16 Department of Dermatology, Venerology and Allergology, University of Leipzig Medical Center, Leipzig, and; 17 Fraunhofer Institute for Translational Medicine and Pharmacology ITMP, Immunology and Allergology, Berlin, Germany

**Keywords:** anaphylaxis, occupational, allergy, drug, venom, food, latex

## Abstract

Background: Anaphylaxis is a systemic allergic reaction that is potentially life-threatening. Occupational anaphylaxis is an anaphylaxis that occurs in an occupational context. In this position paper, we propose diagnostic criteria for occupational anaphylaxis and provide an overview of the current state of knowledge in terms of prevalence, triggers, prevention, and management. Results: The most common triggers of occupational anaphylaxis include Hymenoptera venoms, followed by food and drugs. Chemicals, bites or contact with animals (mammals/snakes/insects) and natural rubber latex are far less common. Occupations at risk for occupational anaphylaxis are therefore beekeepers, outdoor workers, or those who handle food as well as healthcare workers. The route of contact, intensity, and frequency of exposure, type of allergen, and the simultaneous occurrence of co-factors determine the clinical manifestation. A detailed medical history is required to confirm the diagnosis of anaphylaxis and to identify the trigger. Both skin tests and the determination of specific IgE are recommended, but only very few commercially available and quality-tested allergens are available that can be examined using both test methods. Preventive measures are based on avoiding further exposure or, if necessary, replacing a working substance. A written emergency plan and the prescription of an adrenaline autoinjector as well as instructions for its use are mandatory. Allergen immunotherapy is recommended for systemic Hymenoptera venom allergy. Depending on the national healthcare systems, patients with occupational anaphylaxis must be reported to the accident insurance. Conclusion: Occupational anaphylaxis is very rare. We recommend educational measures and generally standardized recording of occupational anaphylaxis for occupations with an increased risk of anaphylaxis.

## Introduction 

Anaphylaxis is a severe, acute, systemic hypersensitivity reaction characterized by the rapid onset of multi-organ symptoms, mediated predominantly by immunoglobulin E (IgE) [1]. Most frequent elicitors of anaphylaxis are Hymenoptera venoms, foods, and drugs [2]. Diagnosis is based on clinical signs following exposure to a known or potential allergen [1]. The European Academy of allergy and Clinical Immunology (EAACI) as well as the World Allergy Organization (WAO) define it as a serious life-threatening generalized or systemic hypersensitivity reaction [3]. Since different diagnostic criteria for anaphylaxis exist [3, 4] those established by Sampson et al. [4] (Table 1) are used for this paper. 

Occupational anaphylaxis can be defined as anaphylaxis resulting from elicitors and triggering factors relating to a particular work environment [6, 7] but there are no clearly defined diagnostic criteria. Thus, classification of clinical reactions as occupational anaphylaxis may be challenging; possible scenarios are summarized in Table 2. In selected cases, an expert opinion may be necessary. 

Elicitors of occupational anaphylaxis can enter the body by inhalation, dermal contact, accidental inoculation, insect sting, or following hand-to-mouth transmission [6]. The main categories of workplace elicitors include food substances, drugs, insect venoms, mammalian and snake allergens, natural rubber latex (NRL), and chemicals [6, 7] (Table 2). Occupational anaphylaxis most often results from exposure to allergens at the workplace leading to sensitization first and subsequently to anaphylaxis. Workers, however, may also be initially sensitized from workplace allergen exposures, but only manifest anaphylactic reactions in non-workplace contexts, e.g., NRL allergy acquired as a healthcare worker and elicited by latex contact at home [6]. Additionally, anaphylaxis due to allergens that are not primarily occupational in origin may occur in the workplace or may be aggravated by occupational exposure (e.g., repeated Hymenoptera venom anaphylaxis at a beer production facility with many wasps). On the other hand, anaphylaxis occurring at the workplace is not always occupational, e.g., if it arises from non-workplace exposures manifesting at work only by chance (e.g., a peanut-allergic person consuming peanuts at the workplace during break time). In a European internet-based survey, 5.9% of food anaphylaxis occurred at the workplace [8]. 

Overall, occupational anaphylaxis is rare. Data from the European Registry of Anaphylaxis involving 1,000 cases indicated that an occupational context was present only in 3.8% of cases [7]. There are reports on a preponderance of females in non-occupational anaphylaxis, and the mechanistic involvement of sex hormones in immune reactions has been postulated [9]. However, recent data on occupational anaphylaxis from the anaphylaxis registry involving 15,748 cases reported a male preponderance in venom anaphylaxis and no gender differences when food was the elicitor challenging this hypothesis [7]. The aim of this paper is to summarize current knowledge on occupational anaphylaxis with regard to prevalence, triggers, prevention, and management. A further aim of this position paper is to provide up-to-date recommendations for physicians in dealing with patients with an urgent suspicion or confirmed diagnosis of occupational anaphylaxis. 

## Diagnostics 

The diagnosis of anaphylaxis relies on clinical signs and symptoms (Table 1). Differential diagnosis has to be considered, i.e., urticaria, asthma, anxiety/panic attack, fainting, choking [6]. For occupational anaphylaxis, the sensitization to an allergen is acquired and/or the reaction is triggered by allergens or conditions with predominant exposure at the workplace and caused in a professional context [7]. It is crucial to identify the causative occupational agent inducing the reaction. For identification, a careful clinical and occupational history is mandatory (Tables 2, 3). In addition, medical records and pre-existing laboratory and allergy test results have to be studied. The patient’s role in the workflow of the occupational setting (work process) and her/his exposure to potential allergens has to be elucidated, including the study of Safety Data Sheets. 

Serum-specific IgE is only available for selected occupational allergens, e.g., food, NRL, beta-lactam antibiotics, or Hymenoptera venom. In some cases, cellular tests, such as the basophil activation test may be helpful. The diagnostic workup is often based on skin prick tests or intradermal tests. Often, commercially available test extracts are not available. In these cases, skin prick testing with raw and home-made materials should be carried out carefully, and non-irritant skin test concentrations have to be sought for, which ideally requires testing of non-exposed controls. Challenge tests may be required if diagnostic doubt remains and are the only method to verify hypersensitivity to nonallergic elicitors [12, 13]. Correlation of test results with patient history is essential, and possible allergens not being related to the workplace should also be considered, i.e., food, drugs. Foods may elicit anaphylaxis both in an occupational setting and independently, e.g., if the food in question is eaten at the workplace. [Table Table4]

## Management 

The acute management of an anaphylactic reaction at the workplace should follow general guidelines on anaphylaxis [1, 3]. A few weeks after all symptoms subsided, the patients should then undergo the diagnostic pathway to identify the culprit agent [1, 6, 12, 14]. When occupational anaphylaxis is suspected, workers should be removed from possible causative exposures at work until fully investigated [6]. After diagnosis, the patient should be educated to avoid exposure to the culprit agent, both at the workplace and outside of work. Patients who have had serious adverse reactions should be carefully instructed about hidden allergens and cross-reactions to other allergens. If reliable avoidance of the allergen cannot be achieved, patients should also be provided with an anaphylaxis action plan and medications for self-treatment (including adrenaline autoinjector) [12, 14, 15]. At the workplace, every measure to prevent further exposure should be carried out, this includes (according to the STOP principle): **S**ubstitution of elicitors, **T**echnical measures, **O**rganizational measures for elicitor avoidance and **P**ersonal safety precautions. Safety procedures at manufacturing plants for producing medications may be effectively limiting contact to medication. A written emergency management plan as well as health and safety education and training on how to use the adrenaline autoinjector is necessary. If allergen avoidance is not completely achievable, the risk should be minimized by avoiding the direct manipulation of the culprit elicitor and/or wearing protecting gloves, clothing, or a face mask. In any occupational setting where there is a worker with a history of previous anaphylaxis, the availability of adrenaline and a person trained in its administration are mandatory [16]. If possible, patients with insect-induced occupational anaphylaxis should be started on venom immunotherapy (i.e., with Hymenoptera venom). 

Depending on the organization of national healthcare systems, patients with occupational anaphylaxis are handled differently in different European countries concerning the provision of therapy and prevention measures as well as compensation. Implemented insurances against work accidents and occupational injuries exist in several countries [17]. In Germany, urgent medical suspicion of occupational anaphylaxis must be reported to the Social Accident Insurance in Germany (Unfallversicherungsträger) [17]. 

## Examples of common occupational allergens 

### Food 

Most reports on food-induced occupational allergy exist on eliciting contact dermatitis, urticaria, and respiratory symptoms, while food-induced occupational anaphylaxis is rare [6, 7] and is reported as single cases or small case series. Sensitization in food-induced occupational anaphylaxis mostly results from inhalation or from contact dermatitis and only rarely from ingestion of the offending food. More recently, repeated bites of certain tick species, have been reported to induce sensitization against galactose-α-1,3-galactose (alpha-GAL), which may result in an anaphylaxis to mammalian (“red”) meat, offal, and related products. In 2018, an alpha-GAL sensitization in a cook was accepted for the first time as an occupational disease in Germany [18]. Data on occupational alpha-GAL sensitization are limited [19]. In 300 forest service employees and hunters from Southwest Germany, 19.3% of the studied persons showed alpha-GAL sensitization with specific IgE ≥ 0.35 kU/L. Of those, 8.6% had a history of delayed meat-induced anaphylaxis [20]. 

Occupational food allergy may also develop due to immunological cross-reactions between non-food allergens and food, e.g., in healthcare workers a sensitization to NRL may lead to anaphylaxis when eating avocado, banana, etc. [8, 21]. Cooks and other workers involved in food processing or food handling who get in permanent contact with foods are reported to be most frequently affected by food-induced occupational anaphylaxis [22]. Accordingly, 3 – 11% of seafood industry (fish, crustacean) employees were reported to suffer from contact urticaria to seafood, which in several cases also resulted in systemic signs and symptoms at ingestion [23]. Table 5 summarizes reports of food-induced occupational anaphylaxis. 

### Drugs 

Reports on occupational drug anaphylaxis are limited [6, 16]. One reason is that the full picture of anaphylaxis associated with the workplace is much less common than rhinitis, asthma, or contact dermatitis. Underreporting is another issue. In addition, exposure to drugs at the workplace by inhalation or direct skin contact during drug production might less commonly lead to sensitization and anaphylaxis as compared to ingestion or systemic application. Occupations at risk include healthcare workers handling drugs, pharmacists mixing medications, and people employed in the manufacturing process (Table 5) [6]. There are several reports on nurses with anaphylaxis when handling penicillin, cefotiam, or piperacillin-tazobactam infusions [40, 41, 42, 43, 44, 45, 46, 47]. A non-atopic nurse experienced repeated anaphylaxis even without direct involvement in drug-specific tasks such as crushing of tablets or preparation of injections [48]. In her case, the causal allergen was identified when non-occupational anaphylaxis occurred after oral cefuroxime intake during a respiratory infection and confirmed by allergy testing. Other cases of non-occupational anaphylaxis have been described when workers with occupational exposure received the implicated beta-lactam as a patient and had no history of past personal exposure [49, 50]. Anaphylaxis developed in four South Korean healthcare workers with latent tuberculosis infection who received a 3-months course of once weekly isoniazid and rifapentine [51]. 

Cases of severe occupational anaphylaxis have been described in a dentist, two nurses, and an endoscopy technician when handling chlorhexidine [52, 53]. It may also occur to polyhexanide, a chlorhexidine-like biguanide antiseptic, which is widely used, for example, in contact lens solutions, wound dressings, pool cleaners, and cosmetics [54]. Medical staff is at increased risk of developing polyhexanide sensitization. So far, IgE-mediated anaphylactic episodes to polyhexanide associated with disinfecting wound care and following the use of moist toilet paper that contained polyhexanide as a disinfectant have been described [54, 55]. Individuals with known chlorhexidine allergy may be at risk for anaphylactic reactions to polyhexanide due to cross-reaction or co-sensitization [55]. Occupational anaphylaxis to povidone iodine has been described [56]. 

Chloramine-T is commonly used as a sterilizer, antiseptic, disinfectant, and chemical reagent. A female patient showed generalized itchy erythema, dyspnea, and vertigo 15 minutes after treating a burn by cooling in water with added chloramine-T [57]. She had been probably sensitized during her cleaning activities at a butchery. Chlorocresol and chloroxylenol in cleaning products were attributed to occupational anaphylaxis in a women working as a nurse [58]. 

Anaphylaxis to the fiber laxative psyllium has been reported for non-occupationally ingested psyllium in two nurses with a prior history of occupational exposure, both had previously reported rhinitis when handling psyllium [59]. 

Immunotherapy allergens are a potential trigger for occupational anaphylaxis, as highlighted in the case of a technician with known allergic rhinitis and sensitization to timothy grass who developed rhinoconjunctivitis, urticaria/angioedema, and hypotension after a needlestick while preparing immunotherapy vials of timothy grass extract [60]. 

A final-year anesthesiology resident developed anaphylaxis after cutaneous exposure to succinylcholine. Intradermal testing was strongly positive for succinylcholine and all other neuromuscular-blocking drugs and elicited a widespread erythematous rash and anaphylactic shock [61]. The anesthesiologist could continue his profession without further reaction in the following 12 months by avoiding operating rooms with a high usage of neuromuscular-blocking drugs and by wearing personal protective equipment. Occupational exposure to quaternary ammonium compounds in hairdressers and cleaners was reported to be associated with anaphylaxis during general surgery when different neuromuscular-blocking agents (rocuronium, suxamethonium, cisatracurium, atracurium, mivacurium) were applied [62]. 

There was debate about the risk of anaphylaxis induced by coronavirus vaccines, as two employees of the National Health Service (NHS) in England developed severe allergic reactions after having received vaccination with BNT162b2 vaccine against COVID-19 (coronavirus disease 2019) [63, 64]. However, meanwhile millions of healthcare workers were vaccinated with different COVID-19 vaccines and the risk of occupational anaphylaxis seems to be low [65]. A predisposing factor seems to be history of anaphylaxis [63, 66, 67, 68]. Polysorbate skin testing was not identified as being useful for detecting people at risk for anaphylaxis [68, 69]. 

The increasing use of cannabis for medical purposes in some countries and associated occupational exposure in the growth, cultivation, handling, and dispensing of the cannabis plant has raised concerns about health risks [70]. Until now, there are no reports on occupational anaphylaxis, but it has been stated in a review that up to 20% of individuals with allergic reactions to cannabis may also show anaphylactic-like reactions [71]. [Table Table6]

### Small chemicals 

Low-molecular-weight chemicals are capable of eliciting immunological or non-immunological immediate responses [6, 16] after transdermal or inhalant exposure [73, 74, 75] (Table 7). Diagnostics to detect an IgE-mediated mechanism against a chemical remain case-specific, i.e., the diagnostic procedure usually is not commercially available nor properly standardized [76, 77, 78, 79]. Diagnosis often relies on resolution of symptoms after allergen avoidance. In the agriculture sector, fungicides and insecticides are of relevance. A greenhouse worker suffered from anaphylactoid reactions – contact urticaria as well as tight chest and throat – after skin and/or inhaled exposure of chlorothalonil, a common fungicide used for agricultural and horticultural purposes [74]. In the chemical sector, case reports can be found on metal salts and various chemical compounds eliciting anaphylaxis. A welder reported urticaria, angioedema, and bronchospasm associated with occupational exposure to chromium vapor [80]. A ceramic decorator first developed eczematous skin lesions on the backs of her hands and forearms and subsequently generalized urticaria with angioedema and general fatigue after using blue paint containing cobalt chloride [76]. A 26-year-old process operator exposed to iridium chloride at an electrochemical factory that manufactures titanium anodes developed respiratory tract symptoms and contact urticaria [75]. Benzonitrile is mainly used as an intermediate for rubber chemicals, solvents and, above all, lacquers. A worker presented with contact urticaria syndrome with malaise, nausea, dizziness, sweating, dyspnea, and hypotension with tachycardia while rearranging poorly packaged benzonitrile at her workplace [81]. Hexafluorophosphates are uronium/guanidinium salt coupling reagents used in the biochemical process of solid-phase peptide synthesis. A female researcher reported chest tightness, coughing and sneezing, and urticaria with angioedema while working with hexafluorophosphates in a peptide synthesis laboratory [82]. In another case, a laboratory worker developed anaphylaxis and contact urticaria due to occupational airborne exposure to powdered hexafluorophosphates [83]. Polyethylene glycols (PEG) are used as excipients and solvents in many topical or systemic drugs, cosmetics, hygiene products, and in industrial areas. A painter developed generalized urticaria and angioedema when he was exposed to paints that contained PEG 4000 [79]. 

Anecdotal cases of anaphylactic reactions at the workplace include an operating room nurse after contact to methyl methacrylate [84] and a medical assistant in a family practice office after an assault with sprayed perfume [85]. A nurse was exposed to cinnamate while handling a range of fragranced products, after which she developed urticaria, a burning sensation in her head, and abdominal symptoms [86]. The mechanism of the immediate-type reactions to fragrances is still largely unclear. 

In the personal care sector, ammonium persulfate and potassium persulfate are often used in hair-bleaching formulas and hair dyes to accelerate the bleaching process and thus reduce the amount of peroxide used [77, 87]. Persulfates are also used in the chemical, metal, textile, and pharmaceutical industries, as well as in the photographic, food and especially cosmetic industries [88]. Hairdressers are particularly at risk, where immediate-type symptoms with contact urticaria, angioedema, rhinitis, sneezing, cough, dyspnea, and wheezing due to ammonium persulfate [88] and potassium persulfate have been described [77]. In this context, the occupationally acquired sensitization to these two persulfates can cause anaphylaxis for the first time outside the workplace, as for example in the case of a hairdresser during dental treatment due to the persulfates in the dental cement [89]. Hairdressers and beauticians also have an increased risk of sensitization to hair dye in their workplace. Eleven hairdressers with occupational immediate-type skin and respiratory symptoms attributed to oxidative hair dyes were reported; one of them suffered an anaphylactic reaction with nausea, abdominal pain, respiratory distress, urticaria, and edema after using an oxidative hair dye containing 2,4-toluylenediamine [90]. 

In the textile industry, a wool and cotton dyer suffered dyspneic attacks accompanied by diffuse urticaria during weighing dyes and dyeing woolen and cotton yarns with Lanasol Yellow 4G, an α-bromoacrylamide reactive dye [91]. 

### Hymenoptera venom 

The prevalence of Hymenoptera venom allergy in adults ranges from 0.3 to 7.5% in Europe. Studies carried out in bee-keepers and forestry workers showed a prevalence of occupational anaphylaxis due to Hymenoptera venom of 2 – 3.4% [16]. Recent data from the Anaphylaxis Registry demonstrated the impact of venom allergy in work-related anaphylaxis by showing that the vast majority of all of the registered cases were caused by insects (83%); interestingly, yellow jackets were far more often detected as the culprit insect than honeybees (70 vs. 17%) [7]. 

There are two families of Hymenoptera with clinical importance: the bees (honeybees, bumblebees) and the vespids (yellow jackets, hornets, wasps). With climate change, allergy to stings of the invasive species of fire ants may also become of relevance in Europe [92]. Even though Hymenoptera stings are more prevalent during leisure activities, there are professional sectors that are more often exposed and carry a higher risk of recurrent sting events. Occupational groups particularly at risk for Hymenoptera venom allergies typically include beekeepers, forestry workers, individuals working in greenhouses, farmers, gardeners, landscapers, firefighters, bakery shop assistants as well as construction workers or drivers (especially those operating open vehicles) and outdoor workers active in areas with high insect activity [6, 93]. 

Beekeepers and their family members are heavily exposed to honeybee stings and are thus at very high risk of becoming allergic; therefore, they are considered an important population for the study of venom allergy. A large number of simultaneous stings in 1 year may sensitize a person and then be followed by single-sting anaphylaxis. The greatest risk of anaphylactic reactions was during the first years of beekeeping, suggesting that spontaneous desensitization occurs later. On the other hand, beekeepers stung more than 50 times in a season have been reported not to develop any allergic reaction, and up to now there is still no in vivo or in vitro parameter that would give a positive proof of a high risk for anaphylactic reaction [94, 95]. Some authors report that individuals who have experienced large localized reactions in the past may exhibit systemic symptoms upon subsequent stings and they suggest to provide them with an emergency kit as well as an attentive follow-up [96]. Although bumblebee venom allergy through natural exposure is very rare, it has to be considered for instance in greenhouse workers, who may develop bumblebee venom anaphylaxis, because in greenhouses, bumblebees are used for the pollination of plants. The bumblebee venom anaphylaxis is associated with the length of work history and total number of bumblebee stings [97, 98]. 

After a clinically relevant anaphylactic reaction, diagnostics should focus on detecting the culprit insect and initiate venom immunotherapy [99]. Component-resolved IgE diagnosis is recommended [100, 101, 102, 103]. 

According to the literature, a maintenance dose of 100 µg venom administered during venom immunotherapy, as the only available disease-modifying treatment, can prevent a systemic reaction in the majority of re-stung patients, suggesting a better effect with higher maintenance dose, especially in honey bee venom allergy [99]. 

A recent observational study on individuals exposed to Hymenoptera stings in occupational setting revealed that the majority of patients, with the exception of beekeepers, had no access to an emergency kit. As it was demonstrated that patients after receiving venom immunotherapy did not experience severe systemic reactions following insect stings, the significant role of the this therapy is confirmed [52]. 

In Germany, hymenoptera stings during work are work-related accidents and the resulting local and systemic reactions may establish the responsibility of the relevant accident insurance provider, regardless of whether there is an elevated occupational risk or not and it could be categorized as either a workplace accident or an occupational disease. In cases of a workplace accident associated with an allergic reaction, immediate treatment is warranted at the expense of the accident insurance provider. This is the case even when the insect venom allergy pre-existed before the initiation of professional activities or was not acquired due to occupational influences. In situations where the insect venom allergy is clearly a consequence of a workplace accident, all other necessary therapeutic procedures, including allergen immunotherapy are also covered by the accident insurance provider (e.g., in Germany, occupational disease numbers 4301 and/or 5101 of Berufskrankheitenverordnung; see also management section) [104]. 

### Animal contact/bites 

Animal bites from insects, mammals, or snakes as well as contact to animals was associated with occupational anaphylaxis [105] (Table 8). Contact urticaria, respiratory symptoms, and, rarely, other systemic symptoms are mainly reported from workers having contact to laboratory animals [6]. An approach to primary prevention of laboratory animal allergy was published [105]. Severe immediate-type reactions elicited by different insects apart from Hymenoptera (e.g., caterpillar) may become manifest in outdoor workers. Occupational anaphylaxis was reported after contact to *Thaumetopoea pityocampa* but not to *Th. processionea*, yet. Occupational anaphylaxis caused by snake venoms or snake antivenoms are rare and are mainly reported from non-European countries. 

### Natural rubber latex 

NRL is used to manufacture gloves, tires, condoms, balloons, rubber boots, mattresses, swim caps, catheters, vial stoppers, etc. [121]. NRL exposure can take place either through direct skin/mucous membrane contact or via inhalation. Healthcare workers but also hairdressers, cleaners, housekeeping personnel, food-service workers, as well as workers in NRL processing plants are at risk of developing occupational anaphylaxis to NRL [122, 123]. Individuals allergic to NRL may also experience immediate-type reactions due to cross-reactivity with a variety of fresh fruits, vegetables, and nuts in up to 50% [124, 125]. In the mid-1990s, up to 17% of employees in the health service were sensitized by prolonged direct exposure to powdered NRL gloves as well as by aerogenic exposure to NRL protein-contaminated cornstarch powder [124]. Sensitization is facilitated by high frequency and long duration of glove use, skin irritation by wet work, impaired barrier function in atopic individuals as well as previous or current hand dermatitis, the latter allowing latex proteins to penetrate impaired skin in a considerable higher amount compared to healthy skin [126, 127]. In the meantime, by banning powdered NRL gloves and by introducing synthetic gloves, the rate of new cases of skin and respiratory diseases caused by NRL has fallen by over 80% [124, 128, 129]. Anaphylaxis due to NRL allergy is now relatively rare, but severe reactions have been reported. In 2022, a female patient-care assistant with known NRL allergy experienced a severe, biphasic anaphylactic reaction within minutes after entering her ward decorated with rubber balloons [130]. In a current report from Thailand from 2023 [131] the case of a female physician who suffered from NRL allergy at her workplace following occupational exposure and two latex anaphylaxis episodes during medical and surgical procedures was described. Further cases of severe anaphylactic reactions to latex in healthcare workers have been published [20]. An originally occupationally acquired NRL allergy, e.g., in the healthcare sector, can also be the cause of anaphylactic reactions under non-occupational circumstances. A nurse with skin manifestation using NRL gloves suffered from anaphylaxis during a gynecological examination by a physician using NRL gloves [132]. Eating or preparing fruits or vegetables with protein epitopes similar to NRL has been reported to induce anaphylactic reactions in healthcare workers with a primary NRL sensitization [32]. In a cross-sectional descriptive study conducted in a rural hospital in South Africa [133] with 144 participants, the prevalence of latex allergy was 8.3%, of whom 8.3% had experienced anaphylaxis. Workers with compromised skin and especially atopic individuals should not wear NRL-containing gloves to primarily prevent sensitization. In secondary prevention, a “NRL-free” strategy should be followed [122, 134]. [Table Table9]

## Conclusion 

According to published evidence, in contrast to other milder manifestations, such as urticaria, asthma, or contact dermatitis, occupational anaphylaxis is rare. Therefore, mostly case reports or smaller case series are available. However, recent data from the anaphylaxis registry showed that 3.8% of anaphylaxis cases were attributed to an occupational allergen [7]. The vast majority of these occupational anaphylaxis cases were caused by Hymenoptera venom, while food and drugs were rare elicitors. Anaphylaxis triggered by NRL has now been reported in exceptional cases, only. With regard to professions, beekeepers were most frequently affected (43% of cases), followed by gardeners, farmers (28%, respectively), and individuals working in the food sector, e.g., in restaurants, in baker/pastry, and cooks (11%) [7]. While data indicate an increasing impact of food allergy in occupational anaphylaxis, latex allergy decreases and is much less important as an elicitor of allergic reactions including anaphylaxis than in the 1990s. In patients with venom anaphylaxis, an occupational factor should always be considered. Clinicians also should not forget that the world is changing and so are elicitors of anaphylaxis. For example, invasive species like fire ants, change in eating habits, and novel foods or drugs may gain importance also in occupational anaphylaxis [92]. Workers may develop occupational anaphylaxis not only at work, but also outside the work environment if exposure to the same or to a cross-reacting allergen occurs. Prevention measures in patients with a history of occupational anaphylaxis should include training in allergen avoidance and eliminating the allergen as completely as possible. In cases where allergen contact cannot be safely avoided by moving a worker to an unexposed area at the same or a different company, patients should be equipped with emergency medications including adrenaline autoinjectors and a written emergency management plan. They should receive safety education and training. In occupations at greater risk of occupational anaphylaxis, surveillance and awareness campaigns are recommended. In occupational Hymenoptera allergy, venom immunotherapy should be started [12]. Since underreporting is assumed, we call for awareness, documentation, and registration of occupational anaphylaxis in registries. Our twelve main recommendations are summarized in Table 9. 

## Authors’ contributions 

RT, MW, TW, KB, JR: concept, diagnostics, management, RT, MW, UJ, JZ: food, HD: small chemicals, BW, DW, GH: hymenoptera, AB, MR: latex, KB, GW: drugs, RT, MW,TW, KB, UJ, JZ, DB, GW and all other authors contributed to discussionand revision and gave their consent to the final draft. 

## Funding 

None. 

## Conflict of interest 

RT received research support from Sanofi-Genzyme and the Hautnetz Leipzig/Westsachsen e.V. as well as fees for lectures and consultations from AbbVie, Allmirall, ALK-Abello, LeoPharma, Novartis, Pfizer, Sanofi-Genzyme, Viatris and support for congress visit from CSL-Behring; all outside this paper. She is head of working group “Anaphylaxis” of the German Society for Allergy and Clinical Immunology (DGAKI). 

MW reports support for consultancies, lectures, and other scientific activities from ALK-Abelló Arzneimittel GmbH, Abbvie, Eli Lilly, Mylan Germany GmbH, Bencard Allergie GmbH, Novartis AG, Biotest AG, Sanofi-Aventis Deutschland GmbH, HAL Allergie GmbH, DBV Technologies S.A, Aimmune Therapeutics UK Limited, Regeneron Pharmaceuticals, Inc, Stallergenes GmbH. 

AB reports support for lectures, consultancies, and project funding by Abbvie, Almirall, Amgen, AstraZeneca, Biofrontera, Blueberry Therapeutics, Celldex, Centogene, Escient, Galderma, Genentec, Incyte, Jasper, Leo, Lilly, Phavaris, L’Oreal, Novartis, Regeneron, Sanofi, Shire, Takeda. 

HD reports support for consultancies, lectures, and other scientific activities from LEO Pharma GmbH, Novartis Pharma GmbH, and Stallergenes GmbH, not concerning the contents of the manuscript. 

GH reports personal fees for consultancies or lectures from Abbvie, Allergopharma, ALK-Abelló, Biotest, Eli Lilly, Leti, Novartis, Sanofi, all outside of this work. 

UJ has received hotel accommodation and meals for a lecture and for leading a workshop organized by ALK Abello. The fee went to the organization, the RCB. In addition, another hotel night and dinner were recently provided by ALK Abello. Her research on molecular allergology is funded by the Federal Ministry of Education and Science, the Federal Ministry of Food and Agriculture (BMEL), the German Research Foundation, and the Kanert Foundation, all outside the topic of this article. The Federal Ministry of Technology, Economics and Technology has funded their research on assays for the detection of anti-drug antibodies via the AiF-ZIM program. 

LK reports grants and/or personal fees from Allergopharma, MEDA / Mylan, HAL Allergie, ALK Abelló, LETI Pharma, Stallergenes, Quintiles, Sanofi, ASIT Biotech, Lofarma, Allergy Therapeut., AstraZeneca, GSK, Inmunotk, and Cassela med outside the submitted work; and memberships: AeDA, DGHNO, German Academy of Allergology and Clinical Immunology, HNO-BV, GPA, EAACI. 

MR received fees for lectures from Alk-Abelló Arzneimittel GmbH, Berufsverband Deutscher Baubiologen VDB e.V, Haus der Technik, LetiPharma, ThermoFisher Scientific (Phadia). With regard to the content of this article, there are no conflicts of interest that could arise from an employment relationship, benefits for lectures, or other activities. 

BW reports support for one-day advisory boards and/or lectures and/or other scientific acitivities by ALK-Scherax, Bencard, Biocryst, CSL Behring, Kalvista, Novartis, Sanofi, Takeda, all outside the submitted work. 

OP reports grants and/or personal fees from ALK-Abelló, Almirall S.A., Allergopharma, Stallergenes Greer, HAL Allergy Holding B.V./HAL Allergie GmbH, Bencard Allergie GmbH/Allergy Therapeutics, Lofarma, ASIT Biotech Tools S.A., Laboratorios LETI/LETI Pharma, GlaxoSmithKline, ROXALL Medizin, Novartis, Sanofi-Aventis and Sanofi-Genzyme, Med Update Europe GmbH, streamedup! GmbH, Pohl-Boskamp, Inmunotek S.L., John Wiley and Sons, AS, Paul-Martini-Stiftung (PMS), Regeneron Pharmaceuticals Inc., RG Aerztefortbildung, Institut für Disease Management, Springer GmbH, AstraZeneca, IQVIA Commercial, Ingress Health, Wort&Bild Verlag, Verlag ME, Procter&Gamble, ALTAMIRA, Meinhardt Congress GmbH, Deutsche Forschungsgemeinschaft, Thieme, Deutsche AllergieLiga e.V., AeDA, Alfried-Krupp Krankenhaus, Red Maple Trials Inc., Königlich Dänisches Generalkonsulat, Medizinische Hochschule Hannover, ECM Expro&Conference Management, Technische Universität Dresden, Lilly, Paul Ehrlich Institut (PEI), Japanese Society of Allergy, Forum für Medizinische Fortbildung, Dustri Verlag, all outside the submitted work and within the last 36 months; and he is Vice President of EAACI and member of EAACI Excom, member of external board of directors DGAKI; coordinator, main or co-author of different position papers and guidelines in rhinology, allergology, and allergen-immunotherapy and Associate Editor of the journal(s) Allergy and Clinical Translational Allergy (CTA). 

JR has received honoraria for consulting and lectures from Sanofi, Viatris, Allergika, AbbVie, Pfizer. 

SS reports support for consultancy and lectures from Mylan Germany, DBV Technologies S.A., Aimmune Therapeutics, all outside this paper. 

TW reports support for consultancy, lectures, and other scientific activities from AbbVie, ALK Abello, Almirall, Astellas, Bencard, Galderma, Janssen/JNJ, Leo Pharma, Leti, Lilly, Novartis, Pfizer, Regeneron/Sanofi, Stallergenes. 

TZ reports support for consultations, lectures and other scientific activities by AstraZeneca, AbbVie, ALK, Almirall, Astellas, Bayer Health Care, Bencard, Berlin Chemie, FAES, HAL, Henkel, Kryolan, Leti, Lofarma, L’Oreal, Meda, Menarini, Merck, MSD, Novartis, Pfizer, Sanofi, Sanoflore, Stallergenes, Takeda, Teva, UCB as well as responsible participation in the following organizations: Committee member, WHO initiative ‘Allergic Rhinitis and its Impact on Asthma’ (ARIA), Member of the Board, German Society for Allergy and Clinical Immunology (DGAKI), Head, European Centre for Allergy Research Foundation (ECARF), Secretary General, Global Allergy and Asthma European Network (GA^2^LEN), Member, Committee on Allergy Diagnosis and Molecular Allergology, World Allergy Organization (WAO). 

KB reports support for consultancies, lectures, and other scientific activities from ALK-Abelló Arzneimittel GmbH, Bencard Allergie GmbH, Novartis AG, HAL Allergie GmbH, DBV Technologies S.A, ThermoFisher, all outside of this paper. 

DW, WF, TJ, FR, GW, JZ have no conflict of interest.

**Table 1.** Criteria for the diagnosis of anaphylaxis according to Sampson et al. [4].

**Anaphylaxis is highly likely when any one of three criteria are fulfilled:**	
1.	Acute onset of an illness (minutes to several hours) with involvement of the skin, mucosal tissue, or both (e.g., generalized hives, pruritus, or flushing, swollen lips-tongue-uvula)
**and at least one of the following**	
a.	Respiratory compromise (e.g., dyspnea, wheeze-bronchospasm, stridor, reduced peak expiratory flow (PEF), hypoxemia)
b.	Reduced blood pressure (BP) or associated symptoms of end-organ dysfunction (e.g., hypotension (collapse), syncope, incontinence)
2.	Two or more of the following that occur rapidly after exposure to a likely allergen for that patient (minutes to several hours):
a.	Involvement of the skin-mucosal tissue (e.g., generalized hives, itch-flush, swollen lips-tongue-uvula)
b.	Respiratory compromise (e.g., dyspnea, wheeze-bronchospasm, stridor, reduced PEF, hypoxemia)
c.	Reduced BP or associated symptoms (e.g., hypotension (collapse), syncope, incontinence)
d.	Persistent gastrointestinal symptoms (e.g., crampy abdominal pain, vomiting)
3.	Reduced BP after exposure to known allergen for that patient (minutes to several hours):
a.	Infants and children: low systolic BP (age specific) or greater than 30% decrease in systolic BP*
b.	Adults: systolic BP of less than 90 mmHg or greater than 30% decrease from that person’s baseline

*Low systolic blood pressure for children is defined as less than 70 mmHg from 1 month to 1 year, less than (70 mmHg + [2 × age]) from 1 to 10 years, and less than 90 mmHg from 11 to 17 years.

**Table 2.** Diagnostic criteria for classification of occupational anaphylaxis.

**Sensitization at workplace probable**	**Anaphylaxis at workplace**	**Classification as occupational anaphylaxis**	**Example**
Yes	Yes	Yes	Insect allergy in beekeepers Food allergy in cooks or in workers in food service sector and in food industry
Yes	No	Yes	Drug allergy in healthcare workers (e.g., reaction when receiving antibiotic)
No/unknown	Yes	Yes, if allergen source belongs to typical work material	Food allergy in cooks or in workers in food service sector and in food industry
No/unknown	Yes	Yes, if sensitization at workplace is probable even if allergen source does not belong to typical work material	Insect allergy in forest workers
No/unknown	Yes	No, if allergen contact is attributable to private activities	Food consumption at work during break time

**Table 3.** Selected workplaces with possible elicitors of occupational anaphylaxis.

**Workplace/Occupation**	**Possible elicitors**
Healthcare worker	Drugs, natural rubber latex, chemicals
Beekeeper, gardener, green house worker, sales assistant in bakeries and confectioneries	Insect venom
Food production and processing	Animal and vegetable food (inhalation, ingestion/skin contact)
Hunter, ranger	Alpha-GAL in meat, insect venom
Laboratory worker	Chemicals, animal bites/contact

**Table 4. Table4:** Important aspects for history taking in occupational allergy [10, 11].

**Allergen exposure**	Exposure at the workplace or outside
	Route of exposure
	Frequency of exposure
	Type of allergen
**Potential cofactors**	Physical activity, exercise
	Alcohol
	Stress
	Acute infections
	Concurrent medications (i.e., beta-blocker, angiotensin-converting enzyme inhibitors)
**Predisposing individual risk factors**	Older age
	Increased levels of basal tryptase, mastocytosis
	Uncontrolled Asthma
	Cardiovascular disease
	Sex

**Table 5.** Animal- and plant-derived elicitors of occupational anaphylaxis.

**Allergen**	**Occupation/Workplace**	**Reference**
Animal-derived food allergens		
Sushi	Cook Truck driver	[24, 25]
Seafood	Cook	[22]
Anisakis	Fish monger, supermarket^#^	[26]
Cow’s milk	Dairy industry	[27]
Donkey’s milk	Food laboratory	[28]
Quail egg	Poultry worker	[29]
Oyster	Oyster mushroom farmer	[30]
Red meat/alpha-GAL	Forest service employee, hunter^§^, cattle worker, cook	[18, 20, 31]
Plant-derived food allergens		
Avocado, banana, chestnut, manioc, and others	Healthcare worker with sensitization to natural rubber latex as cross reaction	[21, 32]
Buckwheat	Cook	[33]
Broccoli	Cook	[24]
Chicory	Cook	[34]
Coriander	Spice grinder	[35, 36]
Paprika	Spice and condiment seller	[37]
Sunflower	Sunflower processing worker	[38]
Tamarillo	Carpenter with sensitization to obeche wood as cross reaction	[39]
Wheat flour	Cook #	[24]

^#^Generalized urticaria; ^§^no clinical reactions given in detail) [20].

**Table 6. Table6:** Drugs as elicitors in occupational anaphylaxis.

**Group of allergen/elicitor**	**Allergen**	**Occupation/workplace**	**Reference**
Antibiotics	Pencillin Cefotiam Piperacillin-tazobactam Isoniazid Rifapentin	Healthcare workers	[40, 41, 42, 43, 44, 45, 46, 47, 48, 49]
Disinfectants	Chlorhexidine Povidone Iodine Polyhexanide Chloramine Chlorocresol/chloroxylenol	Healthcare workers Butchery Carer	[52, 53, 54, 55, 56, 57, 58, 72]
Laxative	Psyllium	Healthcare workers	[59]
Allergen immunotherapeutic	Timothy grass	Healthcare worker	[60]
Coronavirus vaccines	In question; direct complement activation?	Healthcare workers	[63, 64, 66]
Narcotic agents	Succinylcholine Neuromuscular blocking agents (several)	Healthcare worker Hair dresser Cleaner	[61, 62]

**Table 7.** Chemicals as elicitors of occupational anaphylaxis.

**Elicitor/Allergen**	**Application**	**Workplace/occupation**	**Reference**
Chlorothalonil	Fungicide	Greenhouse	[74]
Iridium chloride	Chemical	Electrochemistry	[75]
Cobalt chloride	Paint	Ceramic decorator	[76]
Polyethylene glycols	Excipients, solvents	Painter	[79]
Chromium vapor	Chemical	Welder	[80]
Benzonitrile	Intermediate for chemicals	Chemical industry	[81]
Hexafluorophosphates	Coupling reagents	Laboratory worker	[82]
Methyl methacrylate	Adhesive	Healthcare worker	[84]
Perfumes	Cosmetics	Healthcare worker	[85, 86]
Persulfates	Bleachings	Hair dresser	[87, 88, 89]
Hair/textile dyes (2,4-toluylenediamine/Lanasol Yellow 4G)	Colorings	Hair dresser, textile industry	[90, 91]

**Table 8.** Animals (bites/contact) as elicitors of occupational anaphylaxis.

**Elicitor/allergen**	**Contact**	**Workplace/occupation**	**Reference**
Rodents			
Rat	Bite	Laboratory	[106]
Mouse	Bite	Laboratory	[107]
Rat, mouse	Bites (N = 2)	Laboratory	[108]
Rabbit	Needle injury after contact to rabbit tissue	Research physician	[109]
Snakes			
Different snake venoms	Bites (n = 3)	Outdoor worker	[110]
Viper	Bite	Research laboratory	[111]
* Atractaspis corpulenta*	Death after bite, anaphylaxis suspected (reaction to antivenom was not ruled out)	Mechanical foreman	[112]
* Bothrops* spp.	Bite	Herpetologist^1^	[113]
* Bothrops moojeni*	Bite	Viper farm	[114]
Insects			
* Rhiphicephalus* spp.	Tick bite	Goatherd	[115]
* Bruchus pisorum*	Inhalation of dust of peas infested by *B. pisorum*	Farmer	[116]
* Thaumetopoea pityocampa* ^2^	Skin contact	Pine/oak or resin collectors/ woodcutters Farmers/Stockbreeders Farmers/stockbreeders	[117]
* Thaumetopoea pityocampa* ^2^	Skin contact	Pine-resin worker	[118]
* Thaumetopoea pityocampa* ^2^	Skin contact	Forest	[119]
* Glossina morsitans* ^3^	Bite	Laboratory	[120]

^1^Experts working with amphibians and reptiles; ^2^processionary caterpillar; ^3^tsetse fly.

**Table 9. Table9:** Summarized recommendations for management of occupational anaphylaxis

1.	Improved diagnostic criteria for classification of occupational anaphylaxis have to consider that
a.	sensitization may take place at the workplace or outside the workplace (e.g., Hymenoptera allergy)
b.	clinical reaction may not only manifest at the workplace but also outside the workplace (e.g., drug allergy in healthcare workers).
2.	The acute management of an anaphylactic reaction at the workplace should follow general guidelines on anaphylaxis.
3.	In Germany, urgent medical suspicion of occupational anaphylaxis must be reported to the Social accident insurance in Germany (Unfallversicherungsträger).
4.	Prevention measures in patients with a history of occupational anaphylaxis should include training in allergen avoidance and eliminating the allergen as completely as possible.
5.	Every measure to prevent further exposure should be carried out, this includes (according to STOP principle): Substitution of elicitors, Technical measures, Organizational measures for elicitor avoidance, and Personal safety precautions.
6.	Patients with history of occupational anaphylaxis should be equipped with emergency medications including adrenaline autoinjectors and a written emergency management plan. They should receive safety education and training.
7.	In any occupational setting where there is an employee with a history of previous anaphylaxis, the availability of adrenaline and a person trained in its administration are mandatory.
8.	In patients with venom anaphylaxis, an occupational factor should always be considered.
9.	In occupational Hymenoptera allergy, venom immunotherapy should be started.
10.	Workers with compromised skin and especially atopic individuals should not wear gloves containing natural rubber latex (NRL) – to primarily prevent sensitization. In secondary prevention a “NRL-free” strategy should be followed.
11.	In occupations at greater risk for occupational anaphylaxis, surveillance and awareness campaigns are recommended.
12.	We call for awareness, documentation, and registration of occupational anaphylaxis in registries.
